# The landscape of transcription initiation across latent and lytic KSHV genomes

**DOI:** 10.1371/journal.ppat.1007852

**Published:** 2019-06-12

**Authors:** Xiang Ye, Yang Zhao, John Karijolich

**Affiliations:** 1 Department of Pathology, Microbiology, and Immunology, Vanderbilt University School of Medicine, Nashville, Tennessee, United States of America; 2 Vanderbilt-Ingram Cancer Center, Nashville, Tennessee, United States of America; 3 Vanderbilt Institute for Infection, Immunology and Inflammation, Nashville, Tennessee, United States of America; Wistar Institute, UNITED STATES

## Abstract

Precise promoter annotation is required for understanding the mechanistic basis of transcription initiation. In the context of complex genomes, such as herpesviruses where there is extensive genic overlap, identification of transcription start sites (TSSs) is particularly problematic and cannot be comprehensively accessed by standard RNA sequencing approaches. Kaposi's sarcoma-associated herpesvirus (KSHV) is an oncogenic gammaherpesvirus and the etiological agent of Kaposi’s sarcoma and the B cell lymphoma primary effusion lymphoma (PEL). Here, we leverage RNA annotation and mapping of promoters for analysis of gene expression (RAMPAGE) and define KSHV TSSs transcriptome-wide and at nucleotide resolution in two widely used models of KSHV infection, namely iSLK.219 cells and the PEL cell line TREx-BCBL1-RTA. By mapping TSSs over a 96 h time course of reactivation we confirm 48 of 50 previously identified TSSs. Moreover, we identify over 100 novel transcription start site clusters (TSCs) in each cell line. Our analyses identified cell-type specific differences in TSC positions as well as promoter strength, and defined motifs within viral core promoters. Collectively, by defining TSSs at high resolution we have greatly expanded the transcriptional landscape of the KSHV genome and identified transcriptional control mechanisms at play during KSHV lytic reactivation.

## Author summary

Kaposi's sarcoma-associated herpesvirus (KSHV) is an oncogenic gammaherpesvirus and the etiological agent of Kaposi’s sarcoma and the B cell lymphoma primary effusion lymphoma (PEL). Despite identification of the virus over 20 years ago there is still an incomplete understanding of how many RNAs are transcribed from the viral genome and the location these RNAs are derived from. To fill this gap in knowledge we determined the landscape of transcription initiation on the KSHV genome. Our analyses more than tripled the number of known TSCs and thus viral-expressed RNAs. Furthermore, we identified key sequence features associated with the regulation of viral transcription start sites. This study provides the first transcriptome-wide characterization of KSHV transcription initiation sites as well as a framework for future studies to define functions of novel viral transcripts and viral gene regulatory elements.

## Introduction

Regulated gene expression is an essential process for all eukaryotic cells as well as the pathogens that infect them. A fundamental step in the regulation of gene expression is the initiation of RNA synthesis. RNA Polymerase II (RNAP II) transcription begins with the binding of gene-specific regulatory factors within the core promoter located near the transcription start site (TSS). Sequence elements found within core promoters include the TATA element, BRE (Transcription factor II B recognition element), Initiator (Inr), and downstream promoter element (DPE) [1]. These elements serve as binding sites for subunits of the transcription machinery and their nucleotide composition can impact the efficiency of transcription initiation.

Models of transcription initiation often depict RNA synthesis initiating from a single nucleotide. However, the application of high-throughput 5’-end sequencing technologies, such as Cap Analysis of Gene Expression (CAGE), have demonstrated that most RNAP II promoters have an array of closely spaced TSSs instead of the expected single TSS [2–7]. Thus, promoters can be more accurately described as a distribution of initiation events (transcription start site clusters, TSCs) on a stretch of given nucleotides [5]. Moreover, the nucleotide sequences that comprise the core promoter can influence the distribution of TSSs as well as the expression profile for a given gene. This has led to the classification of animal promoters into two major groups, narrow and broad [8, 9]. For example, more broadly distributed TSSs are correlated with CpG islands and ubiquitously expressed genes, whereas promoters harboring TATA and TATA-like sequences exhibit a narrow distribution of initiation sites and frequently drive expression of tissue-specific genes [4, 10–15].

Viruses employ a parasitic lifestyle thus they exploit many, and in some cases, all, of the host gene expression machineries. The co-option of cellular processes allows viruses to dramatically downsize their genomes, however, it necessitates the incorporation of host cell-specific gene regulatory mechanisms and features that enable the use of such mechanisms. Additionally, the compact nature of viral genomes requires the encoding of viral proteins and RNAs within a relatively small amount of genomic space.

Kaposi sarcoma-associated herpesvirus (KSHV) is a human oncogenic virus and a member of the gammaherpesvirus subfamily [16, 17]. While KSHV infection is generally unproblematic in the healthy adult population, in the context of immunosuppression such as iatrogenic immunosuppression or advanced HIV-infection resulting in AIDS, the virus is associated with several malignancies [18, 19]. In fact, it is estimated that approximately 1% of all human tumors are associated with KSHV infection and the World Health Organization (WHO) has classified the virus as a class I carcinogen [18, 20]. KSHV is the etiological agent of all forms of Kaposi sarcoma [21–23], a complex and highly vascularized solid tumor of endothelial origin [24], as well as the B cell lymphoproliferative disorders multicentric Castleman’s disease (MCD) and primary effusion lymphoma (PEL) [25, 26].

As with other herpesviruses, KSHV displays two distinct phases of its viral lifecycle, latency and lytic infection. During latent infection KSHV exists in a dormant state in which viral gene expression is dramatically restricted such that few viral antigens and no viral particles are produced. In contrast, during the lytic cycle, which is driven by the viral-encoded transcription factor replication and transcription activator (RTA) [27, 28], the full repertoire of viral genes is expressed and new infectious virions are produced. KSHV has a large DNA genome and similar to other DNA viruses has a complex gene organization including overlapping genes and polycistronic mRNAs. The current annotation of the KSHV genome has over 90 genes that encode for viral proteins and both long and small noncoding RNAs [29].

Despite identification of KSHV over two decades ago we still lack fundamental knowledge regarding how viral gene expression is controlled at the level of transcription initiation. Additionally, although several studies have employed RNA-seq to define the transcriptional output of the KSHV genome as well as the temporal kinetics of viral gene expression, it is often difficult to accurately identify TSSs from traditional RNA-seq data alone. Identification of TSSs is particularly important in complex transcriptomes, such as that of KSHV, where there is extensive overlapping transcription of viral genes and the appearance of polycistronic RNAs. To fill this gap in fundamental knowledge we have mapped all transcription initiation events on the viral genome using RNA annotation and mapping of promoters for analysis of gene expression (RAMPAGE) [30]. Leveraging RAMPAGE, we now provide transcriptome-wide nucleotide resolution TSC annotations for viral genes in two widely used models of KSHV infection, namely the iSLK.219 [31, 32] and TREx-BCBL1-RTA [33] systems. We confirm 48 of 50 previously known TSSs as well as identify over 100 novel TSCs in each cell line. Moreover, these analyses identify cell-type specific differences in TSC positions as well as promoter strength, and define sequence elements comprising viral core promoters. Collectively, we greatly expand the transcriptional landscape of the KSHV genome and identify transcriptional control mechanisms at play during KSHV lytic reactivation.

## Results

### Transcriptome-wide identification of KSHV transcription start sites at single-nucleotide resolution by RAMPAGE

Precise promoter annotation is required for understanding the mechanistic basis of condition- and tissue-specific gene regulation. While the transcriptional landscape of KSHV has been characterized by RNA-seq there is still a gap in fundamental knowledge regarding the location of viral TSSs, and thus it has not been feasible to comprehensively define viral promoters and the sequence motifs embedded within them. Moreover, whether there are condition (i.e. latent vs. lytic) or cell type specific differences in TSS usage is not known. To fill this gap in knowledge we took advantage of two well established models of KSHV infection, namely, iSLK.219 and TREx-BCBL1-RTA cells. While iSLK.219 cells are of clear cell renal cell carcinoma origin and thus of lesser biological relevance [34], the iSLK system has proven invaluable to KSHV research as it is routinely used in studies addressing viral gene function through bacterial artificial chromosome (BAC) mutagenesis. iSLK.219 cells are infected with a recombinant virus, KSHV.219, in which GFP, expressed from the elongation factor 1-α promoter, and RFP, expressed from the viral lytic gene PAN promoter, were inserted into the viral genome [31, 32]. In contrast, TREx-BCBL1-RTA cells are a genetically engineered derivative of KSHV-infected B cells isolated from a patient with PEL [33]. A key feature in both systems is the integration of a doxycycline (Dox)-inducible version of the major viral transcription activator RTA. Thus, upon introduction of Dox into the cell culture media KSHV enters the lytic cycle.

**Fig 1:**
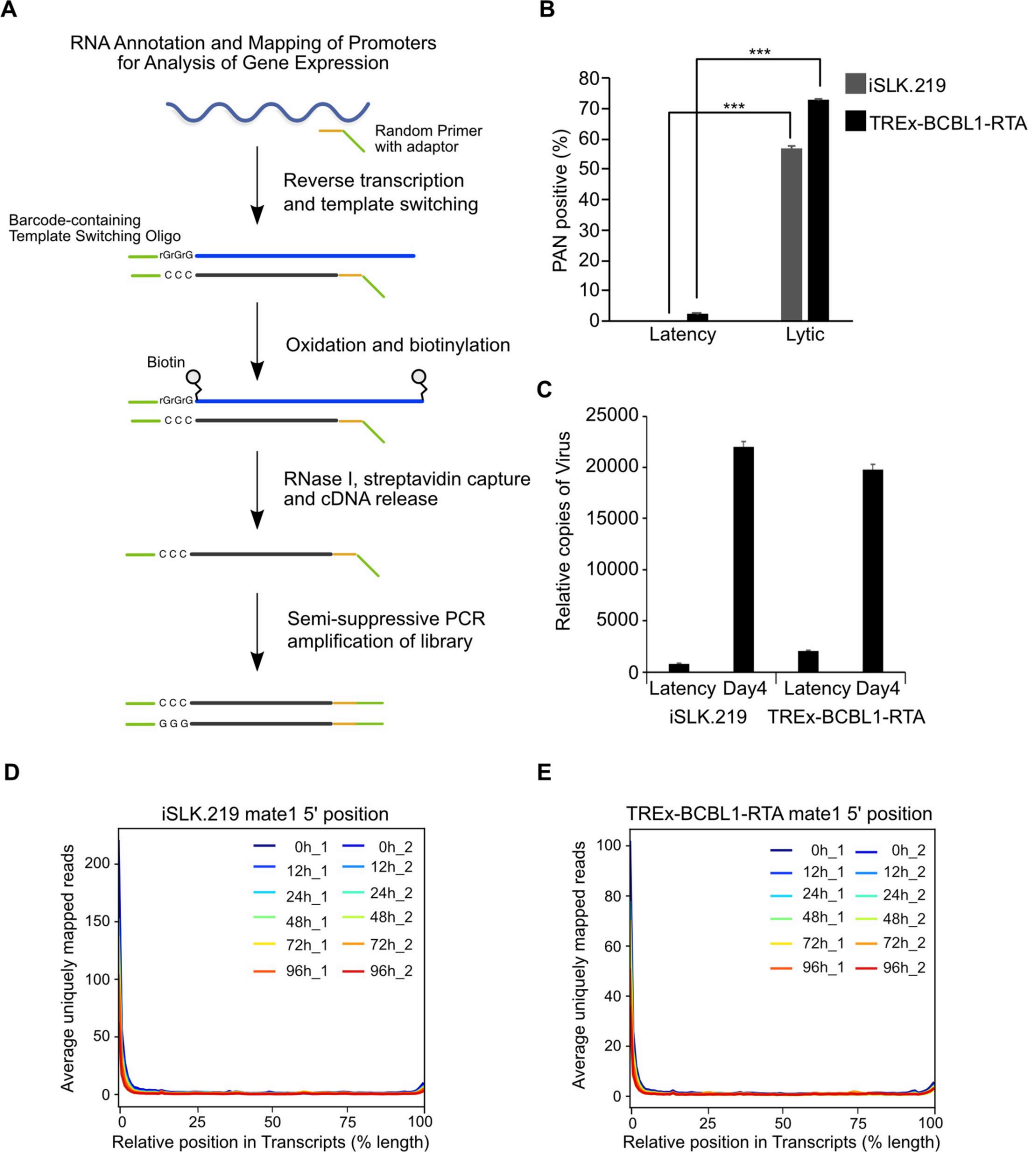
RAMPAGE enables transcription start site identification at nucleotide resolution. (**A**) Schematic illustration of the RAMPAGE method. (**B**) iSLK.219 and TREx-BCBL1-RTA cells were reactivated for 72 h and the percentage of lytic reactivated cells was quantified by PAN RNA FISH-FLOW. (**C**) Virions were isolated from supernatants of iSLK.219 and TREx-BCBL1-RTA cells induced for 96 h and KSHV genomes were quantified by qPCR against ORF52. (**D** and **E**) Average uniquely mapped reads by position for endogenous transcripts, for libraries generated from (D) iSLK.219 and (E) TREx-BCBL1-RTA RNA. Error bars in all panels represent mean±SD from three independent experiments. p Values were determined by the Student’s t test ***, p < 0.001.

Leveraging iSLK.219 and TREx-BCBL1-RTA cells we mapped TSSs transcriptome-wide at nucleotide resolution across a 96 h Dox-induced reactivation time course using RAMPAGE. RAMPAGE, which combines the two orthogonal 5’-selection approaches of template-switching and cap-trapping, enables TSS identification with high specificity through the high-throughput sequencing of 5′‐complete complementary DNAs (Fig 1A). Additionally, an important distinction from CAGE is that RAMPAGE allows for pair-ended sequencing and thus yields extensive transcript connectivity information allowing the annotation of genes with their TSS [30]. Total RNA was isolated from cells in a latent state, or at 12 h, 24 h, 48 h, 72 h, and 96 h post-lytic reactivation and RAMPAGE libraries were prepared. These time points were chosen as we observed >70% of cells in the lytic stage by 72 h post-dox induction as well as a significant increase in virion associated DNA in the culture supernatant (Fig 1B and 1C).

The resultant RAMPAGE sequencing reads were aligned to the human (GRCh38) and KSHV (GQ994935.1) genomes and TSCs were identified by Paraclu [5] and quantified in tags per million reads mapped (TPM). To assess the specificity of our RAMPAGE data for 5′ ends we examined the distribution of the raw TSS 5’ signal over cellular annotated transcripts. Importantly, the metaprofile of signal density clearly demonstrates enrichment near the 5’ end and confirms the specificity of our data (Fig 1D and 1E).

**Fig 2:**
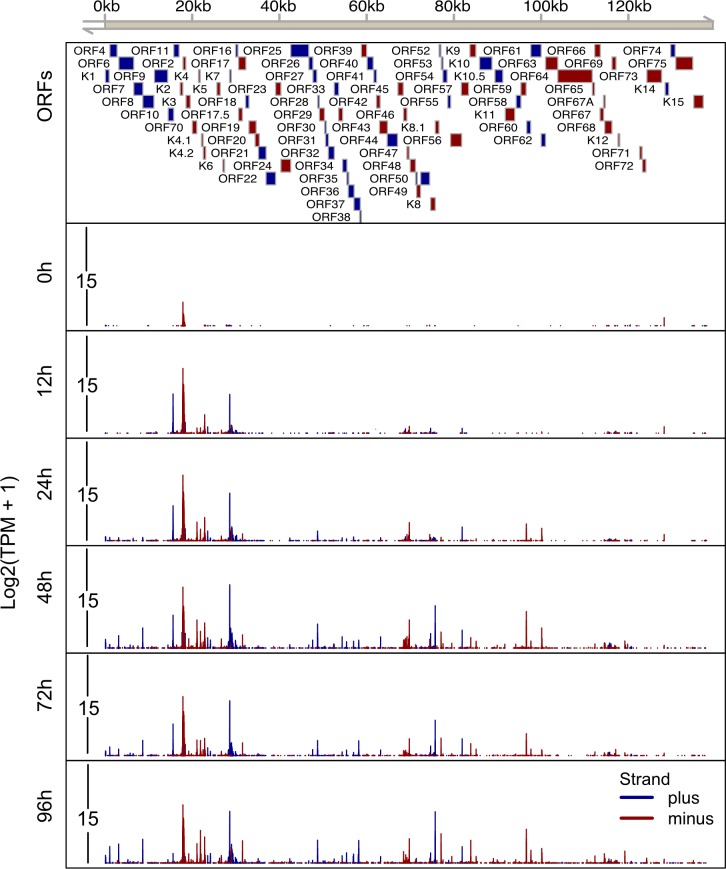
High resolution map of transcription start sites on the KSHV.219 genome in iSLK.219 cells. Total RNA was isolated from iSLK.219 cells at 0h, 12h, 24h, 48h, 72h and 96h post-dox induced lytic reactivation and RAMPAGE libraries were prepared and sequenced. The 5’ signal of read 1, which corresponds to transcriptional start sites, were mapped on the KSHV genome (GQ994935.1). A schematic of the KSHV genome including genomic coordinates of annotated ORFs is depicted on the top horizontal axis. The lower 6 tracks depict the transcripts initiated from every nucleotide across the whole KSHV genome at each time point. The Y axis depicts the log2 transformed TPM value. Blue boxes/lines indicate ORFs/TSSs on the plus strand, red boxes/lines indicate ORFs/TSSs on the minus strand.

**Fig 3:**
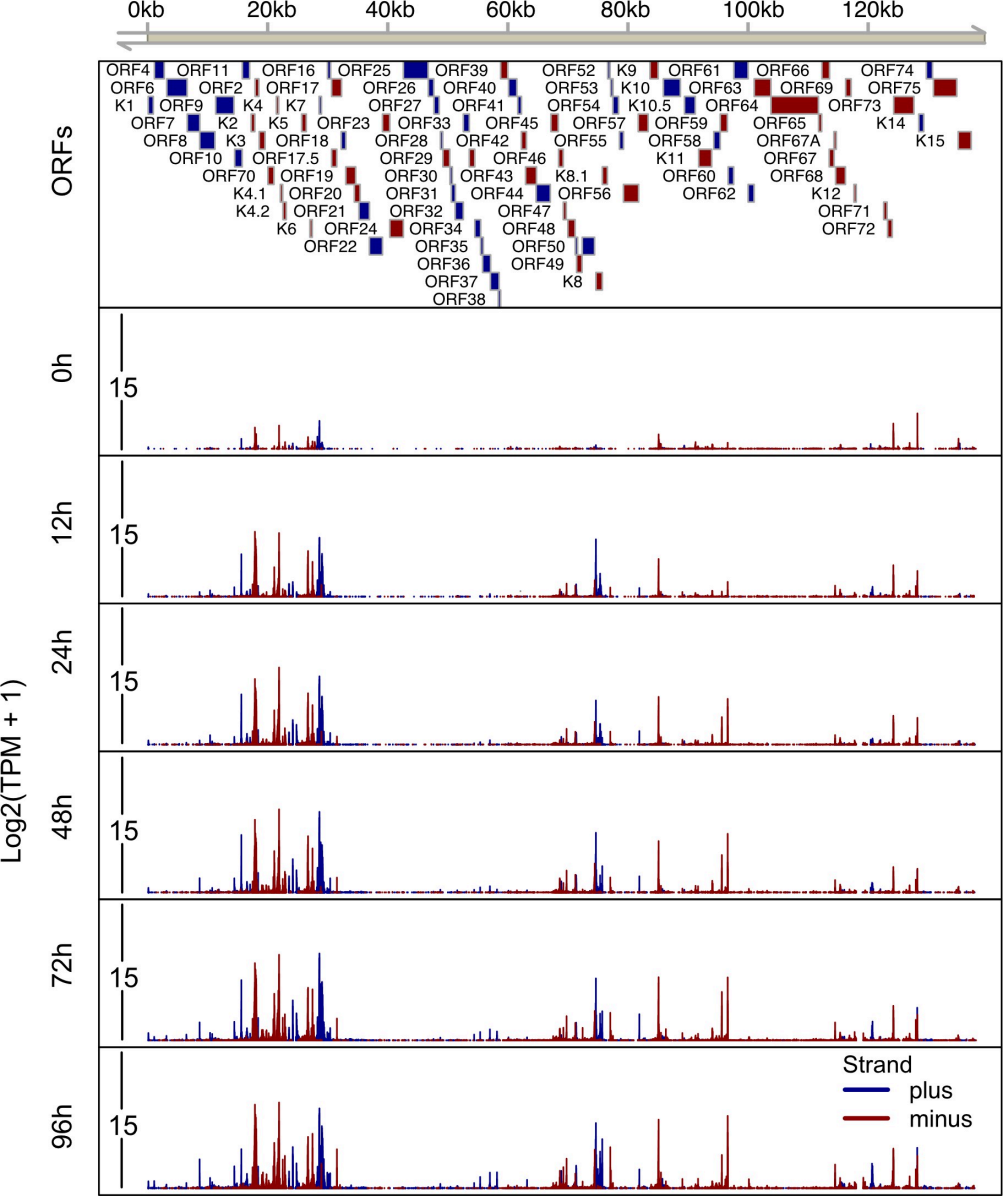
High resolution mapping of transcription start sites on the KSHV genome in TREx-BCBL1-RTA cells. Total RNA was isolated from TREx-BCBL1-RTA cells at 0h, 12h, 24h, 48h, 72h and 96h post-dox induced lytic reactivation and RAMPAGE libraries were prepared and sequenced. The 5’ signal of read 1, which corresponds to transcriptional start sites, were mapped on the KSHV genome (GQ994935.1). A schematic of the KSHV genome including genomic coordinates of annotated ORFs is depicted on the top horizontal axis. The lower 6 tracks transcripts initiated from every nucleotide across the whole KSHV genome at each time point. The Y axis depicts the log2 transformed TPM value. Blue boxes/lines indicate ORFs/TSSs on the plus strand, red boxes/lines indicate ORFs/TSSs on the minus strand.

Within 12 h of Dox addition to the culture media there was an increase in TSSs that continued through the time course (Figs 2 and 3). In total, we identified 164 and 292 TSCs in iSLK.219 and TREx-BCBL1-RTA cells, respectively (Figs 2 and 3, S1 Table). TSSs for 50 viral transcripts have been previous mapped or annotated [27, 29, 35–53]. Within our RAMPAGE data we observed prominent TSSs at the exact position or within a few nucleotides of 48 of the 50 previously identified TSSs, with the exceptions being the TSSs driving ORF17 and ORF42 expression (S1 Fig). We surmise that the slight variations in TSS architecture observed between previous studies and our data is related to the use of histone deacetylase (HDAC) inhibitors to promote KSHV reactivation in the previous reports.

An advantage of RAMPAGE over other 5’-end sequencing technologies is that sequencing is performed in a pair-ended format and thus more information regarding transcript content is present in the data. Leveraging the pair-end information of RAMPAGE we were able to assign many TSSs to ORFs that were previously lacking TSS information (S1 Table). For example, ORF7, ORF10 and ORFK7 were assigned TSSs. This high-resolution map of viral transcription initiation refines the current annotation of KSHV transcriptome, and reveals a much more complex transcriptional landscape than previously appreciated.

### TSCs are enriched in regions of open chromatin

**Fig 4:**
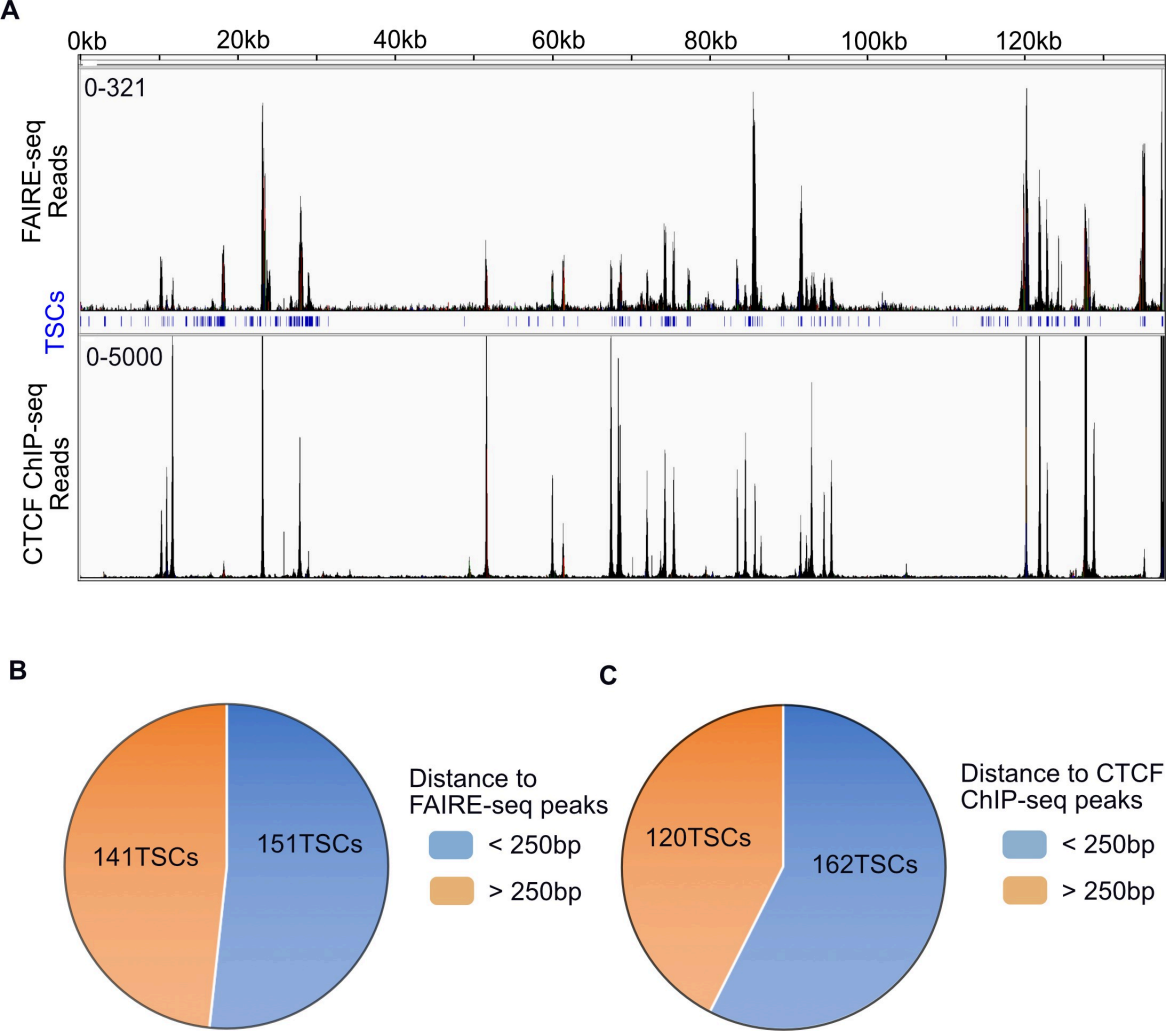
KSHV TSCs are located in regions of open chromatin and CTCF binding. (**A**) Overlap of RAMPAGE identified TSCs with FAIRE-seq and CTCF ChIP-seq signal in TREx-BCBL1-RTA cells. The top coordinates mark positions on KSHV genome, middle blue track shows the position of TSCs defined by RAMPAGE in TREx-BCBL1-RTA cell. (**B**) Pie chart depiction of the number of viral TSCs within 250bp of FAIRE-seq peaks in TREx-BCBL1-RTA. (**C**) Pie chart depiction of the number of viral TSCs within 250bp of CTCF ChIP-Seq peaks in TREx-BCBL1-RTA. The enrichment of TSCs on FAIRE-seq (p < 0.00001) and CTCF ChIP-seq peaks (p < 0.00001) were tested by permutations.

Transcriptional regulation is highly complex and chromatin structure and modifications directly contribute to transcription initiation. The open chromatin landscape of the KSHV genome in PEL has been investigated by formaldehyde-assisted isolation of regulatory elements sequencing (FAIRE-seq) [54]. Previous FAIRE-seq analyses found that regions of open chromatin were not restricted to transcriptionally active loci, but that transcriptionally inactive loci were also nucleosome depleted. Moreover, the transcriptional repressor CTCF occupied the majority of nucleosome depleted regions regardless of transcriptional activity [54]. Having mapped the transcription initiation landscape of PEL cells, we intersected FAIRE-seq and CTCF chromatin immunoprecipitation sequencing (ChIP-seq) data sets with the RAMPAGE defined TSCs from TREx-BCBL1-RTA cells (Fig 4A). Consistent with previous work, we observed that TSCs of both latent and lytic reactivated TREx-BCBL1-RTA cells were highly correlated (p < 0.00001) with regions of open chromatin. Furthermore, we similarly observed enrichment of CTCF at both latent and lytic TSCs (p < 0.00001). In fact, more than 50% of KSHV TSCs are located within 250 bp of a CTCF or FAIRE-seq peak in TREx-BCBL1-RTA cells (Fig 4B and 4C).

TREx-BCBL1 cells have been the subject of extensive investigation regarding the presence and location of various chromatin modifications, including the activating mark H3K4me3 and the repressive mark H3K27me3, as well as the KSHV latent protein LANA and cellular RNAP II [55]. By intersection analysis we visualized the previous ChIP-seq data with our comprehensive TSC map (S2 Fig). Interestingly, although we analyzed H3K4me3 ChIP-seq data from latent TREx-BCBL1-RTA cells, TSCs belonging to both latent and lytic genes are located in close proximity to H3K4me3 signal. However, given that a small percentage of PEL cells are continually undergoing spontaneous reactivation it is possible that the H3K4me3 signal over the lytic genes is derived from this population of cells.

### Evidence for cell-type specific KSHV transcription start site selection

**Fig 5:**
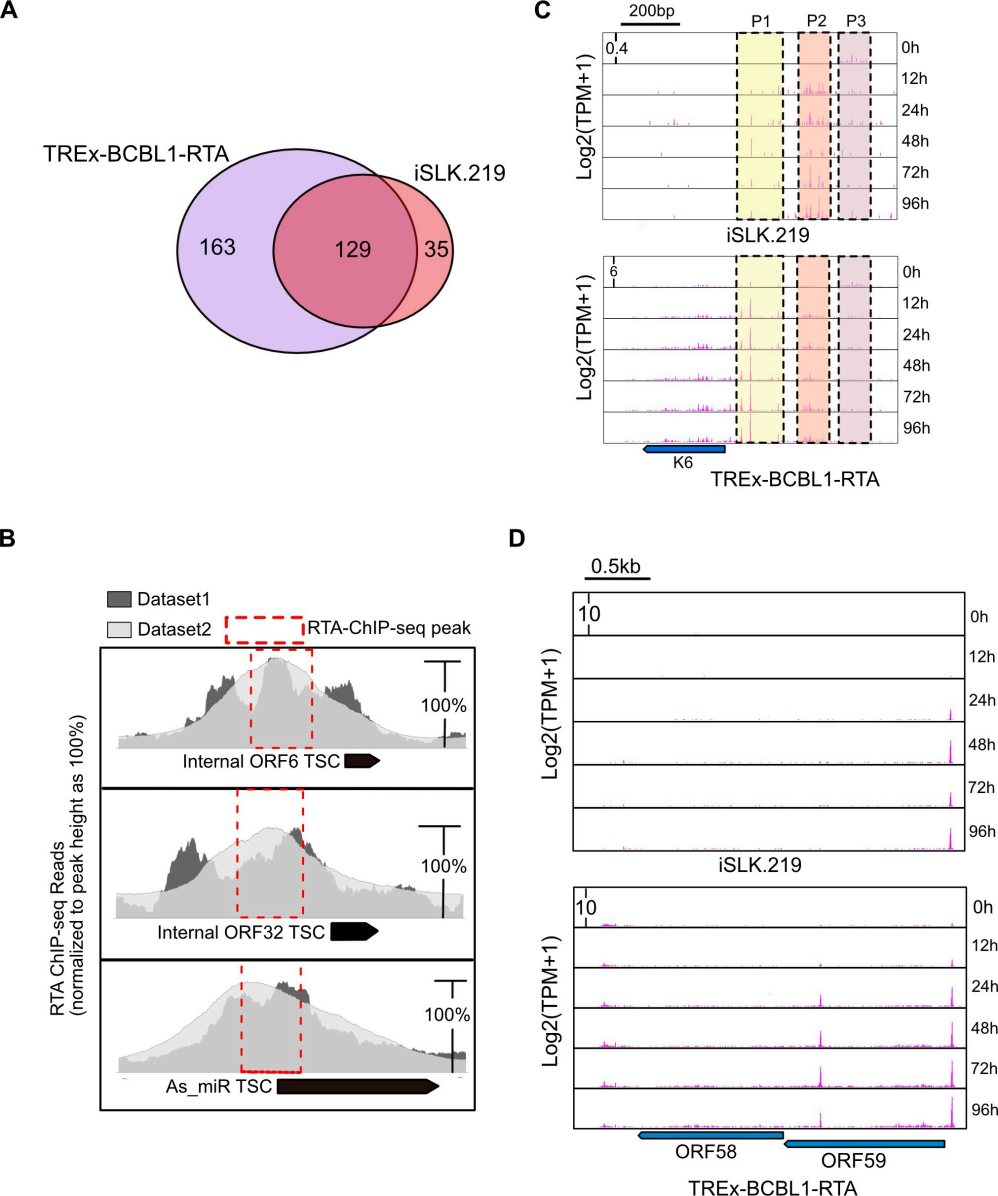
Cell-type specific TSC usage in KSHV-infected cells. (**A**) Venn diagram illustrating the overlap of viral TSCs in iSLK.219 and TREx-BCBL1-RTA cells. (**B**) RTA ChIP-seq read pile associated with three novel TSCs. The peak summit of each RTA ChIP-seq dataset was normalized to 100% for comparison. Black rectangles mark the position of RAMPAGE identified TSCs. Red-dashed boxes represent the location of RTA ChIP-seq peaks identified by MACs2. (**C**) RAMPAGE 5' signal at the K6 locus in iSLK.219 and TREx-BCBL1-RTA cells. The 5' signal on y axis is log transformed TPM value at single nucleotide resolution. The dash-line rectangle marks the primary TSCs (promoters), P1, P2, P3. (**D**) 5’ nucleotide signal of RAMPAGE read 1 associated with ORF58 and ORF59 in TREx-BCBL1-RTA, and iSLK.219 cells. The black bar on the top of the panel indicates the scale, the middle six tracks depict the six different time points post-dox induction. The location of the coding regions for ORF58 and ORF59, which is present on the minus strand, is shown below the panel.

We next sought to determine whether KSHV promoter usage is regulated in a cell-type specific manner. Intersection analysis of TSCs identified in iSLK.219 and TREx-BCBL1-RTA cells revealed that viral gene expression is highly cell-type specific. We identified 129 TSCs were present in both cell lines, we identified 35 and 163 TSCs present exclusively in either iSLK.219 or TREx-BCBL1-RTA cells, respectively (Fig 5A). Importantly, the difference in numbers of TSCs identified is likely not due to sequencing depth as in both iSLK.219 and TREx-BCBL1-RTA a similar number of reads were mapped to the viral genome (S3 Fig). Interestingly, among the 129 TSCs present in both cell-types we identified three novel TSCs that have a corresponding RTA ChIP-seq peak immediately preceding the TSC, suggesting the transcripts are RTA-dependent (Fig 5B). Importantly, the RTA ChIP-seq peaks identified were present in two separate data sets analyzed. In support of a role for RTA in the expression of these TSCs, RTA was capable of activating the expression of a luciferase reporter harboring these genomic fragments as promoters (S4 Fig). While this data does not directly demonstrate the TSCs are RTA-dependent, these data do demonstrate that these genomic fragments harbor the potential to serve as promoters that drive productive transcription initiation.

Inspection of our data uncovered that many immediate early genes, such as K6, tend to be associated with multiple TSCs, all of which are shared between both iSLK.219 and TREx-BCBL1-RTA cells. Previous studies have demonstrated that immediate early genes can be expressed at a low basal level during latency, and are subsequently robustly induced upon lytic reactivation. Given that both multiple shared TSCs are present in both cell-types we hypothesized that basal and induced gene expression may be controlled by different promoters. Indeed, inspection of TSCs that drive expression of K6 uncovered multiple promoters, with a prominent switch in TSC usage upon lytic reactivation (Fig 5C). As shown in Fig 5C, K6 is expressed from three major promoters (P1, P2 and P3). While P3 was preferentially used during latency, usage of P1 and P2 increased substantially upon lytic induction. The conservation of regulated TSC selection for K6 between cell-types suggests this may be an underlying mechanism controlling the proper regulation of K6 expression.

As noted above we also identified cell-type specific TSCs. For example, in TREx-BCBL1-RTA cells we identified 11 ORFs expressed by TSCs only present within TREx-BCBL1-RTA cells. In contrast, we identified a distinct set of 11 ORFs that were expressed from TSCs only identified in iSLK.219 cells. For instance, we identified a TSC for LANA2 (K10.5) in TREx-BCBL1 cells while it was absent from iSLK.219 cells (S5 Fig). This is consistent with a previous report demonstrating LANA2 expression is restricted to PEL and MCD cells [56]. Moreover, in TREx-BCBL1-RTA cells we identified a prominent TSC initiating synthesis of the ORF58 mRNA (Fig 5D), however, in iSLK.219 cells a homologous TSC is not present and ORF58 would be need to be expressed from a bicistronic mRNA ORF58/59 (Fig 5D). However, it is equally possible that ORF58 is not expressed in iSLK.219 cells, and we are unaware of any studies detecting ORF58 protein expression in iSLK.219 cells.

### Lytic reactivation in iSLK.219 and TREx-BCBL1-RTA follows different kinetics

**Fig 6:**
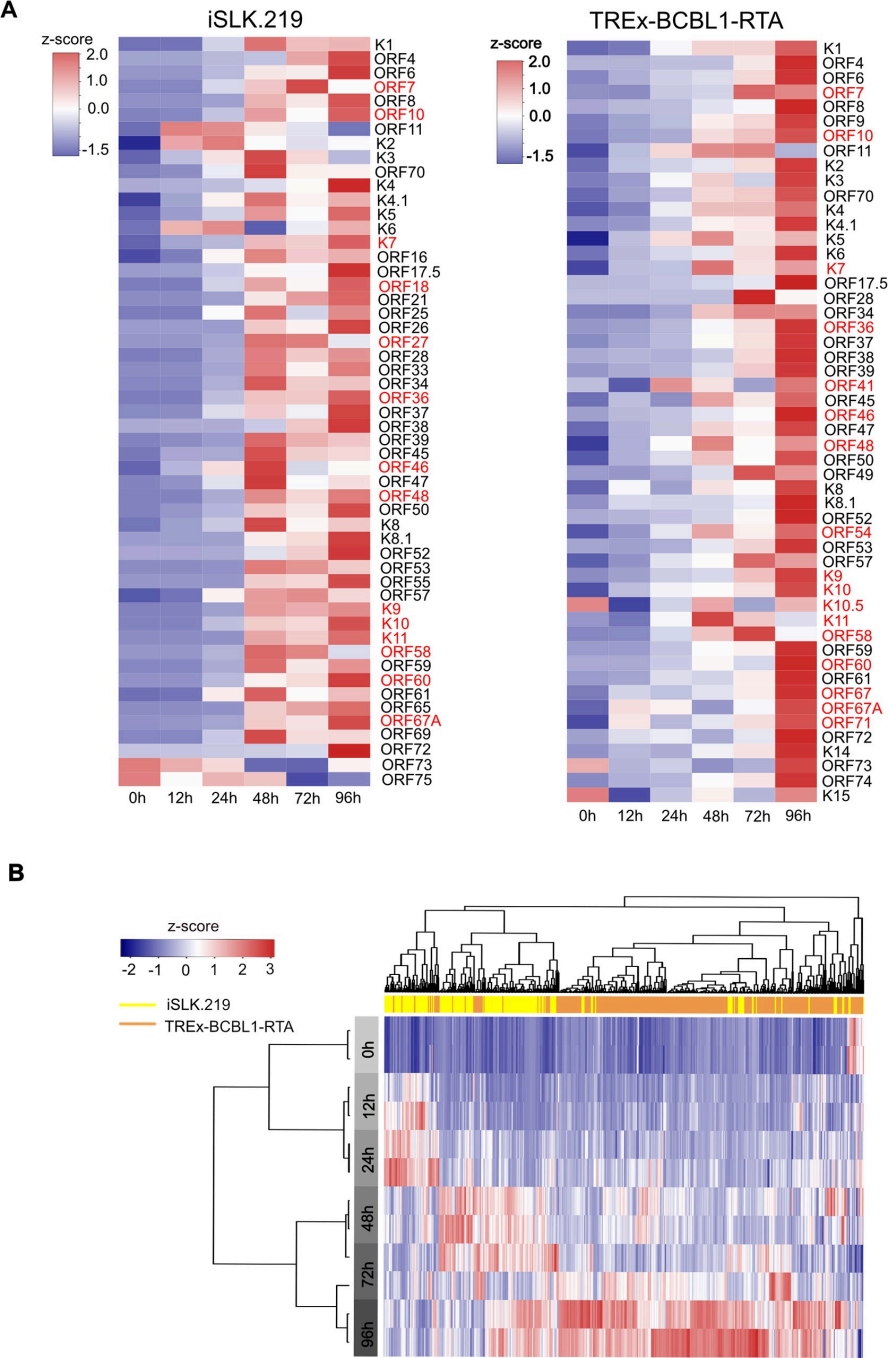
Kinetics of KSHV gene expression leveraging RAMPAGE. (**A**) Heat map illustrating the expression level of known ORFs based on promoter activity at the indicated time points. iSLK.219 cells (left panel) and TREx-BCBL1-RTA cells (right panel). The TSCs of ORFs in red are identified for the first time in this study. (**B**) Unsupervised hierarchical clustering analysis of transcriptional activity of all viral TSCs identified by RAMPAGE. The top horizontal bar indicates the cell lines: yellow and tan represent iSLK.219 and TREx-BCBL1-RTA cells, respectively. The left gray bar indicates the time point of sampling after doxycycline induced lytic reactivation. For all heat maps TPM expressions of TSCs were scaled to Z score before mapping.

In highly complex genomes with extensive genic overlap, 5’-end RNA sequencing is more accurate in measuring transcript expression than traditional RNA-seq as it avoids ambiguous read assignment and transcript length normalization. We thus quantified viral gene expression in both cell lines based on the intensity of the 5’ RAMPAGE signal (Fig 6A). Moreover, since we did not identify prominent TSCs for all ORFs we also quantified viral gene expression by mapping all of the reads directly to the ORFs (S6 Fig). Latency in iSLK.219 cells is extremely tight, and only RNAs belonging to known latent genes were expressed. In contrast, in latent TREx-BCBL1-RTA cells we observed a much broader transcriptional profile, with the promoters of several lytic genes, including ORF11, ORF50, ORFK8, and ORF57, producing mRNAs. The broader expression profile in TREx-BCBL1-RTA cells is consistent with low level spontaneous reactivation in PEL cells.

Unsupervised hierarchical clustering analysis further demonstrated that viral reactivation in iSLK.219 and TREx-BCBL1 cells follows different kinetic programs (Fig 6B). While the activity of most promoters in TREx-BCBL1-RTA cells gradually increases over the 96 h time course, expression from the majority of promoters in iSLK.219 cells peaked between 48 h-72 h (Fig 6A and 6B). Consistent with this analysis, in iSLK.219 cells the number of RAMPAGE reads mapping to the viral genome peak at 48 h, while in TREx-BCBL1-RTA cells this occurs at 96 h post-reactivation (S3 Fig).

### Motif analysis of KSHV TSCs reveals time course related nucleotide usage bias

**Fig 7:**
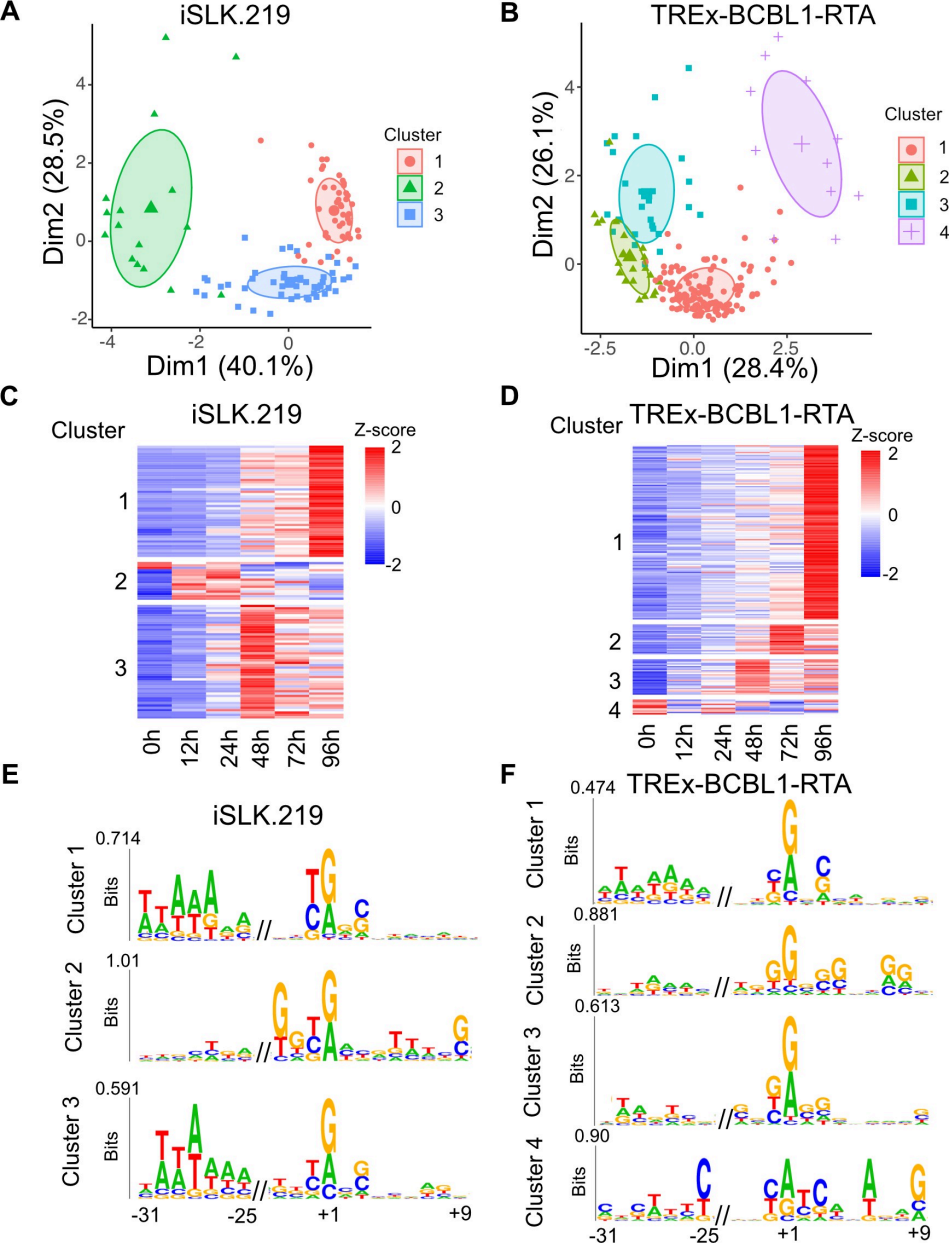
K-means and motif analysis of cis-elements within KSHV promoters. **(A** and **B)** PCA plot of all KSHV TSCs expression for (A) iSLK.219 and (B) TREx-BCBL1-RTA cells. (**C** and **D**) Corresponding heat map of the KSHV TSCs identified for (C) iSLK.219 and (D) TREx-BCBL1-RTA cells. Clusters identified by K-means are shown. (**E** and **F**) Sequences and up and downstream of the MaxTSN within each cluster were extracted and subjected to kplogo analysis. Ultra-short motifs identified in (E) iSLK.219 and (F) TREx-BCBL1-RTA cells are depicted.

Given that unsupervised hierarchical clustering demonstrated distinct kinetic programs we next sought to investigate whether the individual programs were associated with unique core promoter compositions. It has previously been suggested that TSCs within a 50 bp window are under control of the same transcription initiation complex [57], thus we merged TSCs within a 50 bp window into a single cluster and performed K-means analysis on the resulting TSCs. K-means analysis separated the iSLK.219 and TREx-BCBL1-RTA expression profile in to three and four distinct classes, respectively (Fig 7A and 7B). The clusters defined revealed sets of genes with matched peak expression across the time points (Fig 7C and Fig 7D). The identification of variable cluster numbers between iSLK.219 and TREx-BCBL1-RTA transcription programs supports the notion of cell-type specific kinetic programs.

To identify sequence elements associated with the distinct clusters we extracted nucleotide sequences 50 bp upstream and downstream of the maximally expressed nucleotide within each cluster (MaxTSN) and searched for position-specific ultra-short motifs by kplogo analysis. All clusters, with the exception of TREx-BCBL1-RTA cluster 2, display a preference for a Py-Pu dinucleotide at the −1/+1 position and a similar preference has been observed at both mouse and human TSCs [4]. TREx-BCBL1-RTA cluster 2 is also unique in that a GG motif is present at the +7/8 position. Interestingly, when all cellular TSCs are ranked by expression the presence of a downstream GG motif is associated with higher expression (S7 Fig). Consistent with this analysis, cluster 2 displays the highest mean expression value across all time points. While this motif has been previously noted in CAGE data its association with more robust expression was not reported. The molecular basis for this observation is not known although its distance from the MaxTSN is approximately one helical turn of DNA and thus may be phased accordingly with the MaxTSN.

iSLK.219 clusters 1 and 3, which exhibit maximum expression 48 h and 96 h post-induction, respectively, and TREx-BCBL1-RTA cluster 1, which displays maximum expression at 96 h post-induction, all harbor an AT rich region 25–31 upstream of the MaxTSN (Fig 7E and 7F). Moreover, motif analysis using HOMER identified an enrichment of TATA binding protein (TBP) motif within these clusters. However, we are cautious to interpret this as indicating that TBP is involved in the regulation of genes displaying maximum induction at later time points as viral late genes contain a variant TATA motif, TATT, that can also be defined by HOMER as a TBP motif. Thus, it is more likely that the enrichment of TBP motifs within these clusters reflects a combination of TSCs that are driven by TBP and the viral late gene initiation complex.

While neither an AT rich region or TBP motif was found in the other clusters, an initiator (Inr) motif (CA_+1_) is found enriched at TSCs within TREx-BCBL1-RTA cluster four. An Inr motif is similar to a TATA motif in that it can directly recruit the general transcription factor TFIID. However, the Inr recruits TFIID through interactions with TAF1 and TAF2 rather than TBP. The enrichment of Inr motifs in cellular genes that lack TATA motifs has been previously observed and it has been speculated that promoters with an Inr are less dependent on a TATA box and vice versa [58]. Thus, our observation mirrors what has been reported in humans and suggests preferential use of TATA or Inr motifs on viral promoters. Thus, we hypothesize that KSHV uses a combination of sequence elements to recruit TFIID and promote viral gene expression.

We also determined whether the presence of Inr motif was associated with a higher maximum of gene expression. When ranking TSC expression an expanded consensus Inr motif (BBCA_+1_BW) can be found dispersed among the moderately and highly expressed cellular TSCs within iSLK.219 cells (S7 Fig). In contrast, the expanded Inr motif is more commonly identified within higher expressed cellular TSCs in TREx-BCBL1-RTA cells. This suggests that distinct transcriptional control mechanisms and/or preferences may be at play within these two commonly used models of KSHV infection. With regard to viral TSCs, no clear enrichment of the Inr motif is found among genes with a distinct expression profile (S7 Fig).

**Fig 8:**
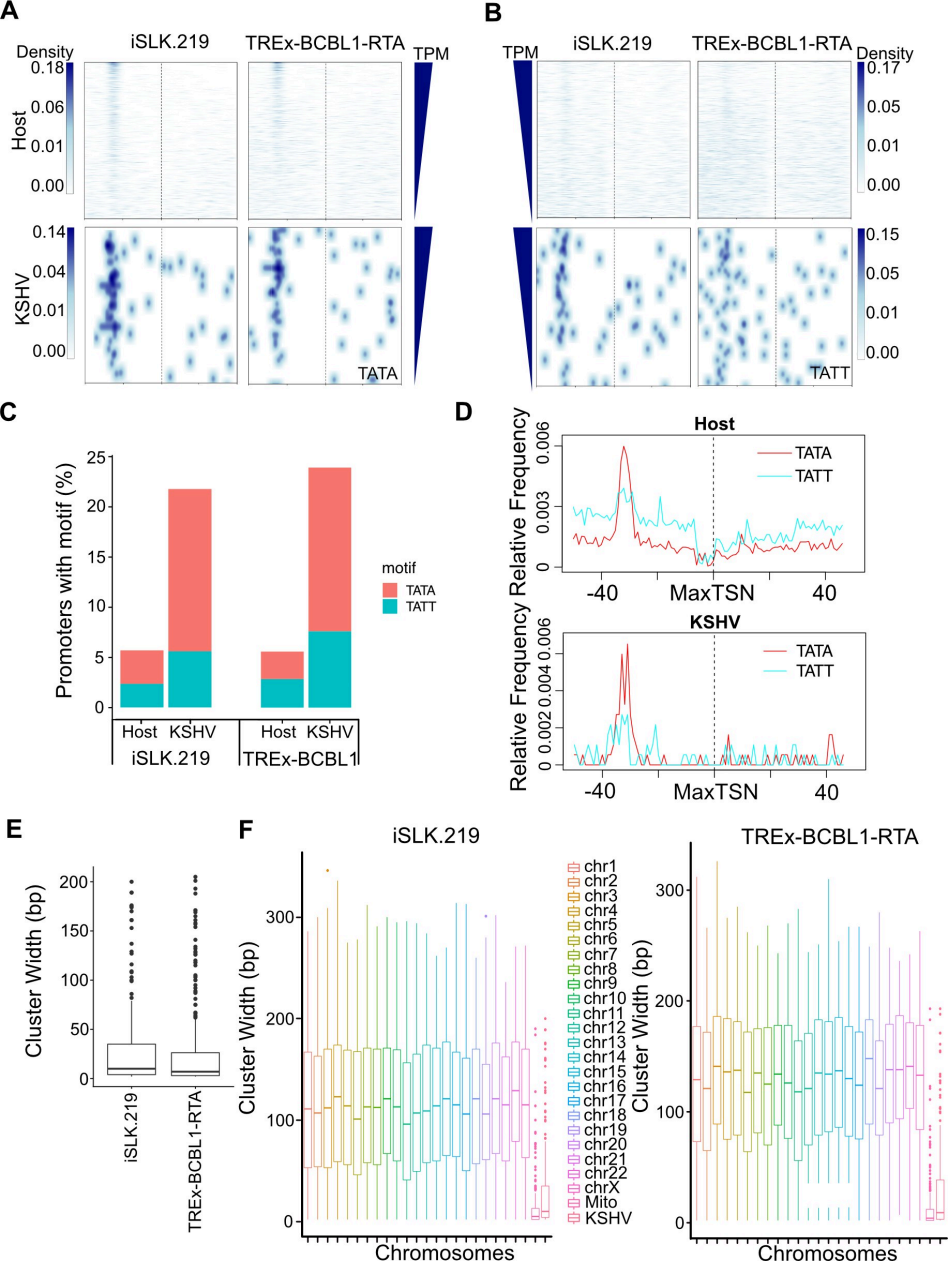
Comparison between host and KSHV cis-element use and TSC architecture. **(A)** Heatmap showing the TATA box motif density 50 bp up- and downstream of MaxTSN in iSLK.219 and TREx-BCBL1-RTA cells. **(B)** Heatmap showing the TATT box motif density 50bp up- and downstream of MaxTSN in iSLK.219 and TREx-BCBL1-RTA cells. In both A and B the TSCs were order by the expression value of MaxTSN. (**C**) Percentage of TSCs with TATA and TATT motifs between 20-31bp upstream of the MaxTSN for host and KSHV. (**D**) Frequency of TATA and TATT motifs found relative to MaxTSN of TSCs from host and KSHV in TREx-BCBL1-RTA cell. (**E**) Cluster width of KSHV TSCs from iSLK.219 and TREx-BCBL1-RTA cells. (**F**) Cluster width of TSCs identified from host chromosomes and KSHV genome for iSLK.219 and TREx-BCBL1-RTA cells.

We also investigated the relationship between the presence of a TATA motif with expression strength. Analysis of the TATA motif density plot clearly shows enrichment of this motif approximately 30 bp upstream of the MaxTSN in both iSLK.219 and TREx-BCBL1-RTA cells (Fig 8A). Interestingly, similar to what was observed for the Inr motif, a TATA motif can be found dispersed among the moderately and highly expressed cellular TSCs within iSLK.219 cells, while it is more prevalent among the highest expressed genes in TREx-BCBL1-RTA cells. With regard to viral TSCs, a TATA motif is not associated with expression.

The TATT motif is a noncanonical TBP-like motif that enables late gene expression via a specialized viral transcription complex [59, 60]. Interestingly, while we observed clear enrichment of this motif approximately 30 bp upstream of the viral MaxTSN in motif density plots, there also appears to be a preference for it upstream of the cellular MaxTSN (Fig 8B). To further investigate this, we first quantified the presence of TATA and TATT motifs within cellular and viral promoters (Fig 8C). Consistent with previous reports we observed TATA motifs within only a small percentage (2~3%) of cellular promoters. Moreover, a TATT motif was also observed in a similar portion of cellular promoters. In contrast, we observed a higher percentage of viral promoters harboring TBP-like motifs. Specifically, we observed a TATA motif within 16% of viral promoters and a TATT motif within 7%. We next calculated the relative frequency of TATA and TATT motifs 50 bp upstream and downstream of cellular and viral MaxTSNs to search for positional enrichment (Fig 8D). Within viral promoters we identified a clear enrichment for these motifs 40–30 bp upstream of the MaxTSN. Interestingly, a second minor enrichment of the TATT motif can be found 25–20 bp upstream of the MaxTSN. The function significance of this second cluster is unclear. With regard to cellular TSSs, the TATA motif is clearly enriched 35–30 bp upstream of the MaxTSN. Unexpectedly, we also observe a peak of TATT motifs at a similar position within cellular promoters. Moreover, there is a clear depletion of this motif immediately upstream of the cellular MaxTSN. We anticipate this suggests that TATT motifs are engaged by cellular TBP for productive transcription and hypothesize that the viral transcription initiation complex may be similarly capable of regulating a subset of cellular genes due to the present of the its preferred binding motif, TATT.

### Viral TSSs exhibit tighter spatial control

The presence of TATA- and Inr-like motifs is primarily associated with tissue-specific or terminally differentiated cell-specific gene expression. Moreover, the presence of these motifs tends to focus transcription initiation events into more well defined clusters. Thus, we compared the breadth of cellular and viral TSCs. The width distribution of viral TSCs is similar between iSLK.219 and TREx-BCBL1-RTA cells (Fig 8E). When compared with the host, we find that the spatial distribution of viral TSCs in both iSLK.219 and TREx-BCBL1-RTA cells is more confined than host TSCs, indicating viral gene expression is under a higher degree of spatial control (Fig 8F).

## Discussion

Comprehensive genome annotations are critical for understanding the biology of all organisms, as well as viruses. Moreover, precise annotations of key gene regulatory features such as TSSs and core promoters, provides the opportunity to define mechanistic features of gene expression regulation. Our transcriptome-wide nucleotide resolution mapping of KSHV TSSs has greatly expanded the transcriptional landscape of KSHV and has identified core promoter sequences associated with TSC architecture and regulation. Moreover, the identification of over 100 novel TSCs in both iSLK.219 and TREx-BCBL1-RTA cells highlights the breadth of the KSHV transcriptome that remains uncharacterized. Future studies must be directed towards understanding the functional significance of the newly defined viral RNAs as they may function as noncoding RNAs or encode proteins that impact the KSHV lifecycle and thus KSHV-associated disease progression.

The high-resolution map of TSSs described here more than triples the number of known transcripts that are expressed from the KSHV genome. However, without full-length RNA-sequencing data it is difficult to fully assess whether the novel TSSs contribute to the expression of yet to be identified RNAs, or whether these extend the 5’-UTRs of known mRNAs. Though analyses here indicate that both are true as we observe novel splice junctions associated with newly discovered TSSs, as well as novel TSSs in which all mate pair reads are associated with a known ORF. Along this line, over 300 previously unknown transcripts were recently identified within the EBV transcriptome through the integration of multiple RNA-sequencing approaches, including CAGE-seq and full-length RNA sequencing [61]. While determining the function of novel transcripts will likely require a detailed description of transcript structure coupled with biochemical assays, we surmise that extension of 5’-UTR sequences impacts gene expression regulation. Indeed, alternative TSS selection has been shown to lead to large differences in translation efficiency of RNA in both yeast and mouse embryonic fibroblasts [62, 63]. Moreover, RNA structures within 5’-UTRs can influence RNA half-live [64, 65]. Thus, in addition to extending the number of RNAs transcribed off the KSHV genome this study also uncovers additional points of entry for post-transcriptional control processes. Furthermore, the use of multiple TSSs to expand the landscape of viral 5’-UTRs appears to be common among viruses as recent studies leveraging CAGE-seq and precision nuclear run-on sequencing (PRO-Seq) have observed multiple TSSs for many EBV and cytomegalovirus (CMV) transcripts [61, 66, 67].

While transcription initiation is often viewed as originating from a single nucleotide, this is an antiquated model. Transcriptome-wide 5’-sequencing methodologies, such as CAGE-seq, PRO-seq, and RAMPAGE, have demonstrated that transcription initiation occurs over a range of nucleotides within promoters [2, 3, 30]. Additionally, low level transcription initiation is also frequently observed within the coding regions of human genes. Given that this has been observed with multiple technologies it is unlikely to be an experimental artifact. Indeed, RAMPAGE analysis on KSHV infected cells reveals that KSHV transcription initiation is similarly structured, with multiple TSSs driving expression of individual genes as well as transcription initiation within protein coding genes. The use of multiple TSSs for an individual protein coding gene enables additional mechanisms of post-transcriptional control of gene expression mediated through the various 5’-UTR sequences. The extent to which KSHV gene expression is regulated via 5’-UTR based mechanisms is unclear, however, our study highlights the need for consideration of these mechanisms when investigating the regulation of viral gene expression.

Using our experimentally defined TSSs we identified unknown similarities and differences in TSC usage between iSLK.219 and TREx-BCBL1-RTA cells. For example, leveraging previously published RTA ChIP-seq data sets we identified three novel TSCs present in both cell types that harbor prominent RTA binding peaks immediately upstream suggesting their expression is regulated by RTA. Moreover, when cloned upstream of a luciferase reporter these genomic fragments confer RTA-inducible luciferase expression. As we noted earlier these data do not conclude that the three novel TSCs are RTA-dependent. However, they do demonstrate the genomic fragments have the potential to drive productive RTA-dependent transcription initiation events. We also identified clear examples where a TSC was specifically used in only one cell-type. For example, in TREx-BCBL1-RTA cells a TSC for ORF58 is identified while in iSLK.219 cells this TSC is absent. While it is possible that that ORF58 is translated from a bicistronic ORF58/59 transcript in iSLK.219 cells, it is equally plausible to hypothesize that ORF58 protein is not expressed there.

Core promoter nucleotide composition is known to influence various aspects of transcription, including the mechanisms of transcription factor recruitment, expression level, and breadth of transcription initiation sites. Leveraging our RAMPAGE mapped TSSs we have defined the nucleotide composition of cellular and viral promoters and defined motifs associated with transcription breadth and strength (Fig 8A, 8B and 8F and S7 Fig). We find that viral promoters more heavily rely on TATA-like motifs than human promoters, and K-means clustering identified a distinct set of viral genes that harbor an Inr motif. TATA-like and Inr motifs are associated with more focused transcription initiation profiles and indeed we discovered that viral promoters drive more focused transcription initiation than cellular promoters. We hypothesize that the high gene density of the KSHV genome, and likely other viruses, necessitates a tighter control on the spatial distribution of transcription initiation events.

During KSHV infection the TATT motif is recognized by the viral TBP mimic ORF24 to facilitate late gene expression [59]. Interestingly, quantification of the TATT motif frequency within cellular promoters identifies a minor enrichment of this sequence approximately 35–30 bp upstream of cellular promoters. TATT can serve to recruit TBP. For example, the mouse a4 IFN promoter contains a well-defined TATT motif [68]. Whether cellular genes that contain this motif are bound by the viral initiation complex is not known although it would be very interesting to investigate this. While it would likely be unfavorable for the virus to activate the expression of an interferon via the viral initiation complex, we hypothesize that cellular promoters that harbor TATT motifs contain sequences that recruit additional factors that are required for their expression. Thus, in this scenario, the binding of the viral late gene complex would not necessarily be activating, but rather could sterically inhibit the recruit of the additional factors and inhibit gene expression.

Our study provides evidence for cell-type specific differences in both TSC usage as well as promoter strength. While the iSLK system has proven invaluable for KSHV community, the transcription initiation profile of the recombinant KSHV.219 virus is distinct from that within PEL cells. While we do identify a much boarder and complex transcriptional profile in iSLK.219 cells than previously known, we identify 130 fewer TSSs within iSLK.219 cells, including the lack of TSCs that facilitate expression of novel RNAs. It is important to note that we cannot ascertain with certainty whether the differences are due to the nature of the viruses, as one is a recombinant virus while the other is the virus that initially infected the BCBL1 cells, or whether KSHV gene expression in iSLK cells is fundamentally different than BCBL1 cells. Additional work is needed to resolve this issue. Moreover, it is formally possible that the bioinformatics thresholds for TSC classification result in a missed TSC in one system. However, if this were the case weakly expressed and spatially dispersed TSCs would be the most susceptible. Nonetheless, our data demonstrate that iSLK.219 and TREx-BCBL1-RTA gene expression programs are distinct.

KS is an endothelial cell derived lesion, and in the future, it will be imperative to determine the viral TSS landscape within endothelial cells. Infection of endothelial cells with virus derived from iSLK.219 cells versus virus prepared from TREx-BCBL1-RTA cells should resolve whether the TSCs landscapes observed here are virus or cell-type specific. Moreover, the KSHV gene expression program is also affected by the environment infected cells are cultured in. For example, during conditions of hypoxia a cluster of genes spanning ORF34-37 are expressed [69]. Application of RAMPAGE to KSHV infected cells cultured in different growth conditions will provide a comprehensive view of how KSHV gene expression is remodeled by the environment and may uncover other unknown viral transcripts.

## Materials and methods

### Cell culture

TREx-BCBL1-RTA (a gift from Dr. Britt Glaunsinger, University of California, Berkeley) was maintained in RPMI 1640 medium (Invitrogen) supplemented with 10% fetal bovine serum (FBS; Invitrogen), 200 μM L-glutamine, 100 U/ml penicillin/streptomycin. iSLK.219 (a gift from Dr. Britt Glaunsinger, University of California, Berkeley) and Human embryonic kidney 293 T (HEK293T) cells (purchased from ATCC) were grown in Dulbecco’s modified Eagle medium (DMEM; Invitrogen) supplemented with 10% FBS. Lytic reactivation of TREx-BCBL1-RTA and iSLK.219 cells was induced by the addition of 1 μg/ml doxycycline (Fisher Scientific).

### Fluorescence in situ hybridization and flow cytometry

Fluorescence in situ hybridization and flow cytometry (FISH-Flow) of PAN RNA in iLSK.219 and TREx-BCBL1-RTA cells was done as previously described [70, 71]. Briefly, cells with indicated treatment were fixed in 4% (vol/vol) paraformaldehyde for 30 min at RT, wash with PBS-FISH buffer (1XPBS, 0.2 mg/ml RNase-free BSA) for twice, and then permeablized with 1X PBS containing 0.2% (vol/vol) Tween-20 for another 30 min at RT. The permeabilized cells were then hybridized with Alexa-Fluor 647 labeled PAN anti-sense oligos (PAN-1, 5’-ACAAATGCCACCTCACTTTGTCGC-3’; PAN-2, 5’-CGCTGCTTTCCTTTCACATT-3’; PAN-3, 5’-GTGAAGCGGCAGCCAAGGTGACTGG-3’) in HB 10% dx buffer (10% (wt/vol) dextran sulfate, 2× saline-sodium citrate (SSC), 10% (vol/vol) formamide, 1 mg/ml tRNA and 0.2 mg/ml BSA) at 37°C overnight. After extensive washing with HBW buffer (2× SSC, 10% (vol/vol) formamide and 0.2 mg/ml RNase-free BSA), cells were analyzed on BD LSR Fortessa instrument. Data were analyzed with FlowJo X software (TreeStar).

### RAMPAGE

RAMPAGE was performed as previously described with minor modifications [30]. RNA was isolated from latent or dox-induced iSLK.219 and TREx-BCBL1-RTA cells by TRIzol (Thermol Scientific) according to manufacture instructions. 5 μg of total RNA from each sample was subjected to ribosomal RNA depletion using NEBNext rRNA depletion kit (NEB) followed by Terminator (Epicentre) treatment. The RNA was purified by RNAClean XP (Agencourt) according to the manufacturer’s instructions and eluted in 7.5 μl H_2_O. 1 μl of reverse-transcription primer (400 μM) and 1 μl of barcoded template-switching oligo (4 mM) were added before denaturation (65°C, 10 min). Ice-cold reverse-transcription mix (7.5 μl Invitrogen first strand buffer, 1.9 μl dNTPs (10 mM), 7.5 μl Sorbitol/Trehalose mix (3.3 M/0.66 M), 1.9 μl DTT (100 mM), 5.6 μl betaine (5 M), 4 μl Invitrogen SuperScript III) was added and the reaction was incubated in a thermal cycler (4°C 10 sec, 22°C 1 min, 42°C 30 min, 75°C 15 min). The RNAs and their associated 5’-complete cDNAs were purified by RNAClean XP, quantified by quantitative PCR and pooled. Pool volumes were adjusted to 40 μl by RNAClean XP precipitation. The oxidization buffer (2 μl 1 M NaOAc pH4.5, 2 μl 250 mM NaIO_4_) was added and the samples were incubated on ice in the dark for 45 min. After quenching the reaction with 2 μl 40% glycerol, 14 μl of 1 M Tris-HCl pH8.5 was added prior to RNAClean XP purification. The samples were eluted in 40 μl H_2_O. Biotinylation was performed by adding the biotinylation mix (4 μl 1 M NaCitrate pH6.0, 13.5 μl 15 mM biotin hydrazide long arm (Vector Labs)) and incubated at room temperature for 14–15 hours in the dark. For RNase I digest, we added the digestion mix (6 μl 1 M Tris-HCl pH8.5, 1 μl 0.5 M EDTA pH8.0, 5 μl RNase I (Thermo Scientific, 10U/μl)) and incubated the samples for 45 min at 37°C, and then 5 min at 65°C. After RNAClean XP purification, the samples were resuspended in 40 μl H_2_O. The biotinylated capped RNAs and their associated 5’-complete cDNAs were captured by streptavidin-coated magnetic beads (Thermo Scientific) for 30 min at room temperature followed by extensive washing: once with wash buffer (WB) 1 (4.5 M NaCl, 50 mM EDTA), once with WB2 (0.3 M NaCl, 1 mM EDTA), twice with WB3 (20 mM Tris-HCl pH8.5, 1 mM EDTA, 0.5 M NaOAc, 0.4% SDS) and twice with WB4 (10 mM Tris-HCl pH8.5, 1 mM EDTA, 0.5 M NaOAc). The cDNAs were eluted by incubation with 65 μl 50 mM NaOH for 10 min at room temperature, and purified with AMPure XP (Agencourt). The cDNAs were then amplified with the KAPA HiFi HotStart PCR kit (KAPA Biosystems). The size selection (~300 to 1000 bp) was conducted with two rounds AMPure XP Cleanup and final libraries were eluted in 20 μl H_2_O. The libraries were run on a DNA HS Bioanalyzer chip for quality control, quantified by quantitative PCR, and sequenced on a NextSeq 500 for PE75 run. Raw RAMPAGE sequencing files can be found in GEO accession # GSE129902. Oligonucleotides used in RAMPAGE analysis are in S2 Table.

### Bioinformatics analysis, sequence alignments

Data processing was performed according to the original RAMPAGE method with minor modifications. Briefly, raw reads in fastq files were demultiplexed and barcodes removed using cutadapt [72]. Trimmed reads were mapped to the human (hg38 assembly) and KSHV (NCBI accession number GQ994935.1) genomes using STAR (V2.6.1b) [73]. Six non-templated G bases on read 1, which were added by reverse transcriptase during the template switch reaction, and 15 bases of read 2, which come from primers used for reverse transcription, were soft clipped in the alignment process. Read pairs that share similar alignment coordinates and an identical reverse-transcription primer sequence were considered PCR duplications and removed. To assess RAMPAGE specificity we calculated 5′- versus 3′-end coverage of uniquely mapping mate 1 reads using the most 5’ nucleotides by deeptools as described in [74]. Reads were mapped to reference transcripts extracted from hg38 and transcripts less than 500 bp were excluded from the analysis [75].

### Identification of transcription start sites clusters and annotation

Start site clusters in mapped RAMPAGE data were extracted using Paraclu [5] with the following parameters (i) a minimum of 10 tags/cluster, (ii) (maximum density/baseline density) ≥ 2 and (iii) 1–200 base cluster length. Consensus clusters between replicates were identified using a custom R script, and subsequently merged into megaclusters by overlap. Coordinates of megaclusters were then applied to calculate the number of tags mapping to each consensus cluster in each sample with Rsubread and normalized for sequencing depth, converting tag counts to tags per million mapped reads (TPM). Assignment of megaclusters to promoters was done with NCBI annotation of KSHV genome GQ994935.1 using following metrics in order: I) megaclusters within 500bp upstream of an CDS were assigned to the gene; II) megaclusters overlapping CDS were annotated as CDS_internal; III) otherwise, megaclusters were considered intergenic. TSS clusters found in iSLK.219 and TREx-BCBL1-RTA cells were considered the same if their coordinates overlap.

### Motif analysis

For motif analysis, TSCs within 50 bp were merged and the maximum expressed nucleotide (MaxTSN) was identified. Sequences 50 upstream and downstream of the MaxTSN were extracted and subjected to motif analysis and pattern density visualization. DNA motif pattern density and position were visualized by R package seqPattern (R package, http://bioconductor.org/packages/release/bioc/html/seqPattern.html), sequences were ordered by MaxTSN expression value (TPM). Transcripts initiating from each of the merged TSS were counted using Rsubread and normalized to the total uniquely mapped reads as TPM (tag per million). The mean expression value of duplicates at each time point were used in the K-means analysis with kmeans function from cluster (R package, https://svn.r-project.org/R-packages/trunk/cluster) and visualized with factoextra, the optimal cluster number K was determined by Average Silhouette Method (R package, http://www.sthda.com/ english/rpkgs/factoextra). The K-means clustered heatmaps were generated with pheatmap (R package, https://github.com/raivokolde/pheatmap). Kplogo was used to search for enriched k-mers at each position [76]. De novo analysis was also performed using HOMER script findMotifsGenomics.pl on nucleotide sequences100bp up- and downstream of MaxTSN using default parameters. hg38 and GQ994935.1 genomes were applied as background [77].

### Data acquisition

CTCF ChIP-seq (SRR503420), FAIRE-seq (SRR965330, SRR965331), and RTA ChIP-seq (SRR5676584, SRR5676585, SRR8324529, SRR8324530), LANA (SRR1030378, SRR1030385, SRR1030386, SRR1030387, SRR1030388), PolII (SRR1030375), H3K27me3 (SRR1030374) and H3K4me3 (SRR1030370, SRR1030371, SRR1030372, SRR1030373) were downloaded from Sequence Read Archive (SRA) use sra-tools (https://www.ncbi.nlm.nih.gov/sra). Processed fastq reads were mapped to GQ994935.1 and GRCh38 genome using default STAR parameters, and peaks identified using MACS2 [78].

### Cloning

Novel RTA-associated TSSs were synthesized as gBlock gene fragments (IDT). gBlock fragments were amplified using KAPA HiFi DNA polymerase (Kapa Biosystems), digested with XhoI and HindIII and cloned into pGL4.28 (Promega). pFLAG-RTA was a kind gift from Dr. Melanie M. Brinkmann (Helmholtz Centre for Infection Research).

### Luciferase assay

HEK293T cells, at 80% confluency, were transfected with 50 ng pGL4.28 luciferase reporter and 450 ng pFlag-RTA (kindly provided by Dr. Kendra Bussey and Dr. Melanie Brinkmann, Helmholtz Centre for Infection Research) plasmid using PolyJet In Vitro DNA Transfection Reagent (SignaGen Laboratories). Cells were collected and used to measure firefly luciferase activity using luciferase assay system (Promega) on a GloMax 20/20 Luminometer (Promega).

## Supporting information

S1 Fig RAMPAGE signal over 10 previously defined TSSs. (TIFF)

S2 Fig Comparison of RAMPAGE defined TSCs to other ChIP-seq datasets. (TIFF)

S3 Fig Number of reads mapping to the KSHV genome in iSLK.219 and TREx-BCBL1-RTA cells. (TIFF)

S4 Fig Luciferase assay for internal ORF6, internal ORF32 and As_miR RTA binding elements. (TIFF)

S5 Fig Comparison of LANA2 RAMPAGE 5’ signal in iSLK.219 and TREx-BCBL1-RTA cells. (TIFF)

S6 Fig Heatmap based on reads mapping to known ORFs. (TIFF)

S7 Fig Pattern density of Inr and GG around MaxTSN (-50~+50). (TIFF)

S1 Table Transcription start sites clusters identified in iSLK.219 and TREx-BCBL1-RTA cells. (XLSX)

S2 Table Oligos used for RAMPAGE and cloning. (XLSX)
