# Supplementary figures and images for: The landscape of transcription initiation across latent and lytic KSHV genomes
Source: PLoS Pathog. 2019 Jun 12;15(6):e1007852. doi: 10.1371/journal.ppat.1007852 (PMC6590836; doi:10.1371/journal.ppat.1007852)

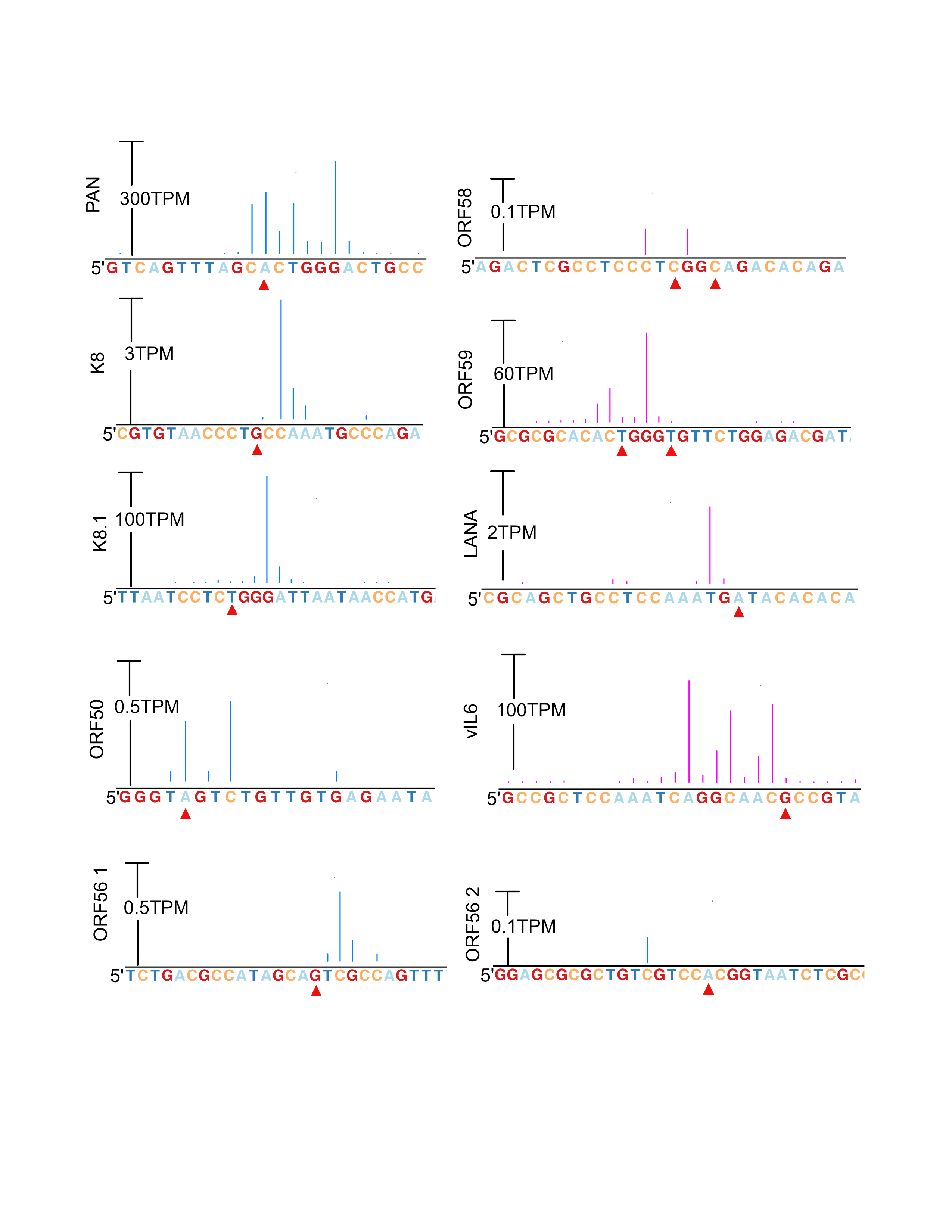

Supplement: S1 Fig — (TIFF) [file ppat.1007852.s001.tiff]

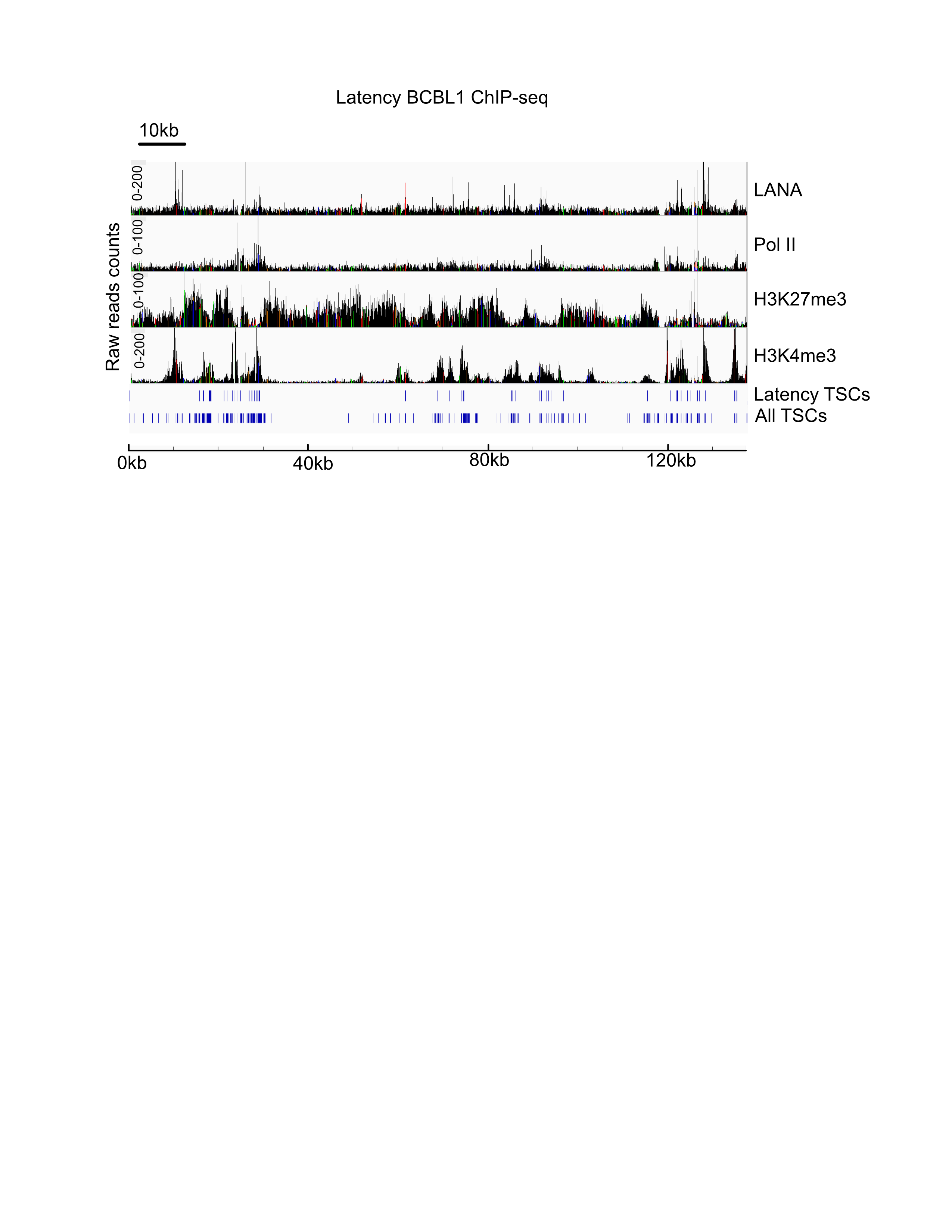

Supplement: S2 Fig — (TIFF) [file ppat.1007852.s002.tiff]

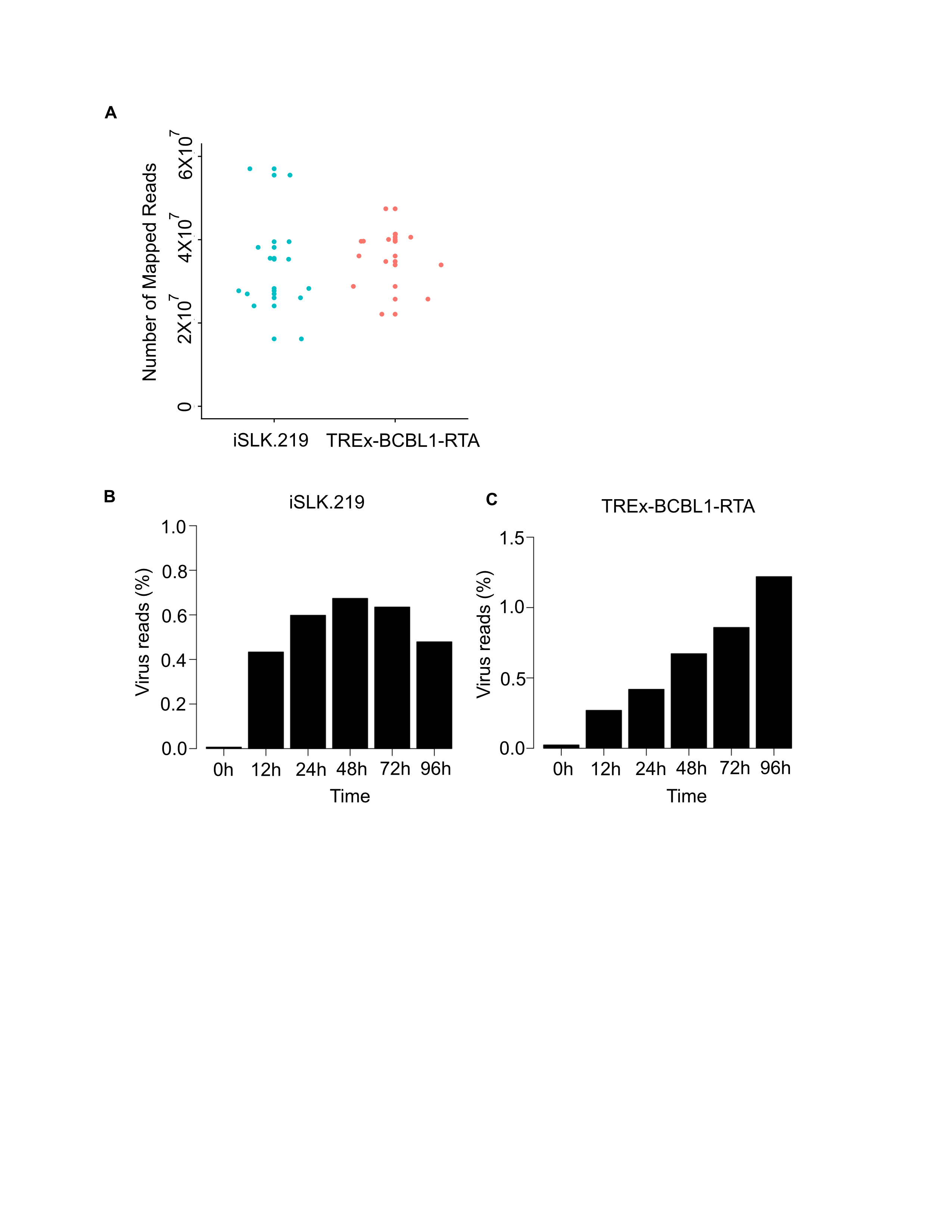

Supplement: S3 Fig — (TIFF) [file ppat.1007852.s003.tiff]

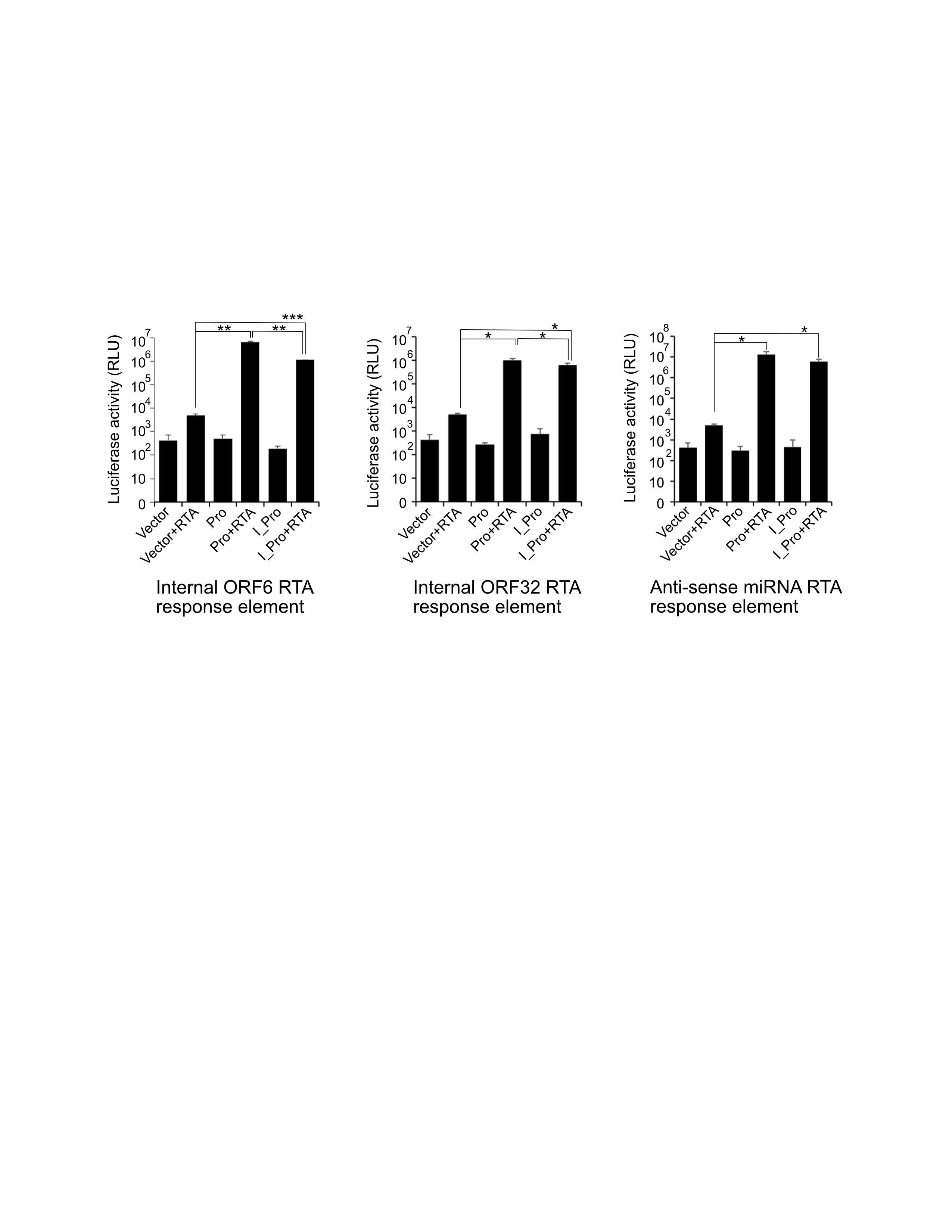

Supplement: S4 Fig — (TIFF) [file ppat.1007852.s004.tiff]

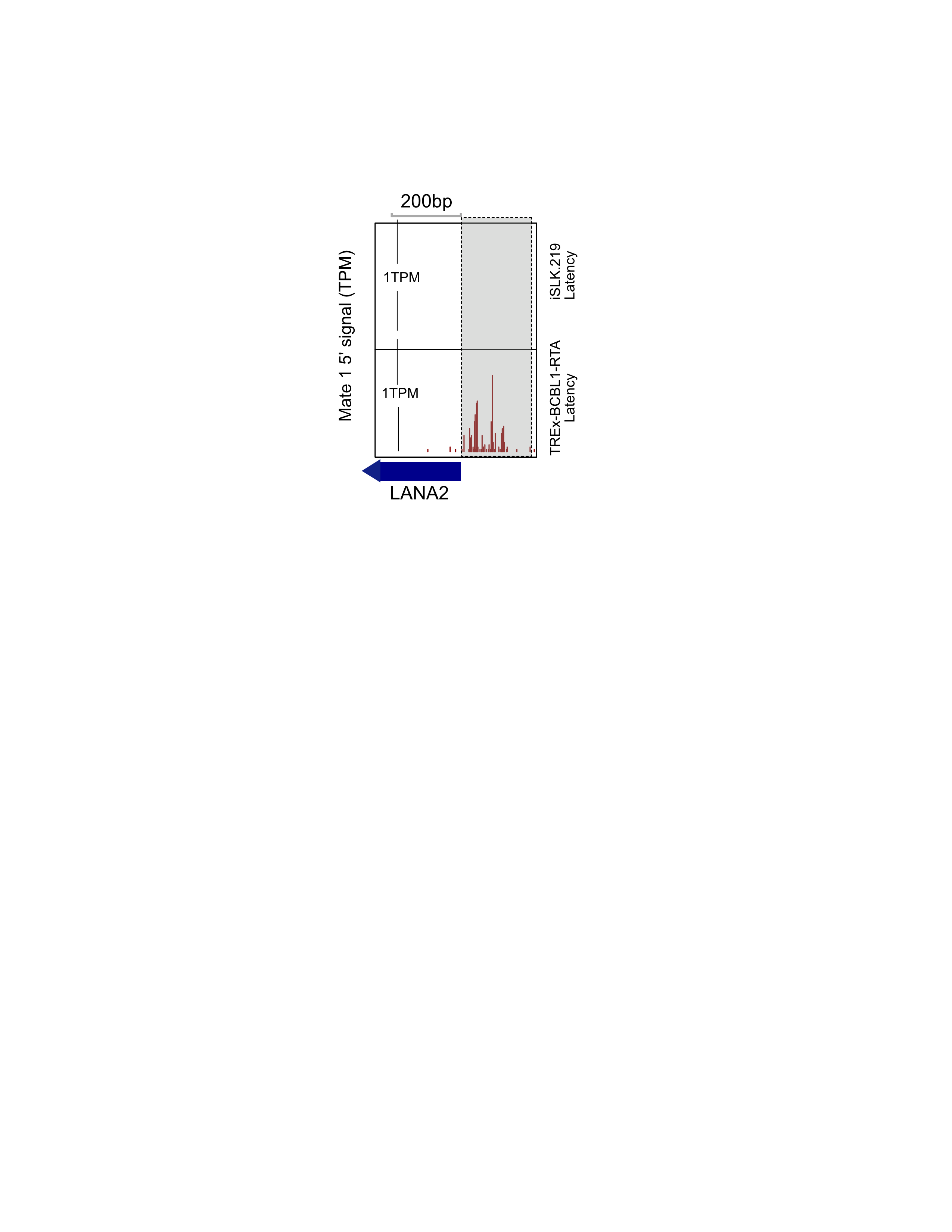

Supplement: S5 Fig — (TIFF) [file ppat.1007852.s005.tiff]

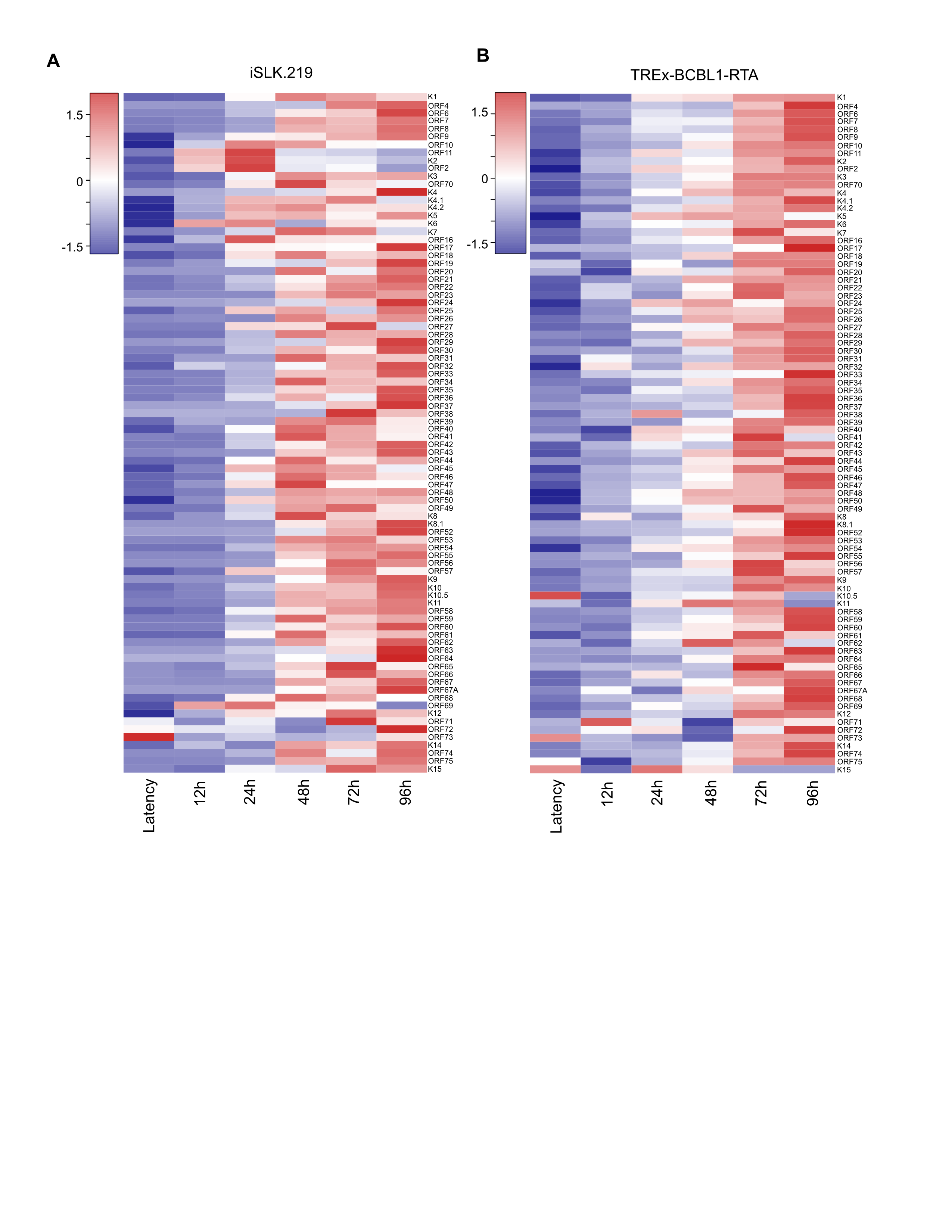

Supplement: S6 Fig — (TIFF) [file ppat.1007852.s006.tiff]

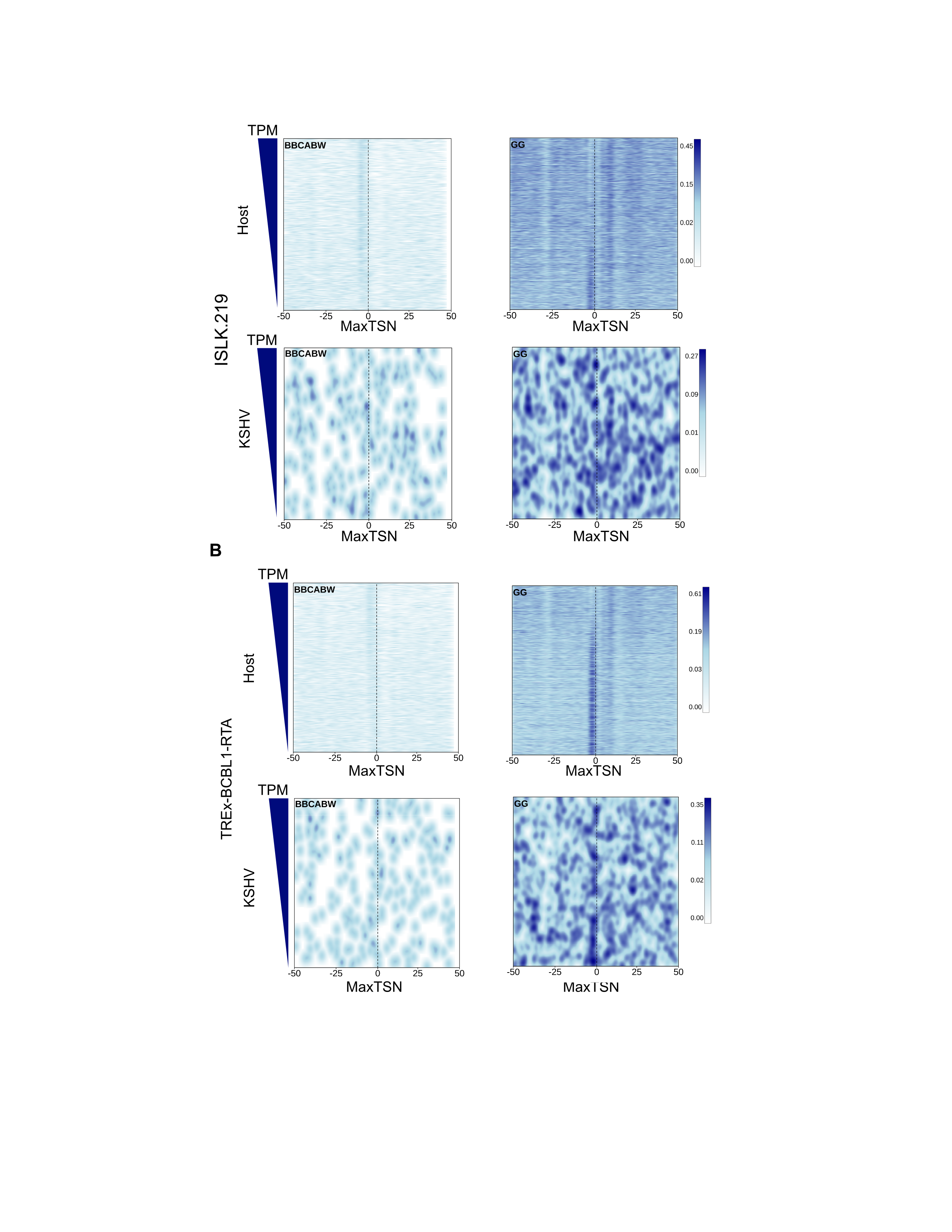

Supplement: S7 Fig — (TIFF) [file ppat.1007852.s007.tiff]
